# Risks of Breast and Endometrial Cancer in Women with Diabetes: A Population-Based Cohort Study

**DOI:** 10.1371/journal.pone.0067420

**Published:** 2013-06-27

**Authors:** Hua-Fen Chen, Ming-Der Liu, Peter Chen, Li-Huan Chen, Ya-Hui Chang, Pei-Chun Wen, Chung-Yi Li

**Affiliations:** 1 Department of Endocrinology, Far Eastern Memorial Hospital, New Taipei City, Taiwan; 2 School of Medicine, Fujen Catholic University, New Taipei City, Taiwan; 3 College of General Education, Hungkuang University, Taichung, Taiwan; 4 Center of General Education, National United University, Miaoli, Taiwan; 5 Department of Gastroenterology, Central Medicine Hospital Group, New Taipei City, Taiwan; 6 Department of Nursing, Yuanpei University, Hsinchu, Taiwan; 7 Graduate Institute of Nursing, Chang Gung University, Taoyuan, Taiwan; 8 Department and Graduate Institute of Public Health, College of Medicine, National Cheng Kung University, Tainan, Taiwan; 9 Department of Dentistry, Buddhist Tzu Chi General Hospital Dalin Branch, Chiayi, Taiwan; 10 Department of Public Health, College of Public Health, China Medical University, Taichung, Taiwan; Baylor College of Medicine, United States of America

## Abstract

**Objective:**

We investigated the overall and age-specific risks of developing breast and endometrial cancer among women with diabetes in a population-based cohort study.

**Methods:**

Women with diabetes (n = 319310) and age-matched controls (n = 319308), selected from ambulatory care claims and beneficiary registry in 2000, respectively were linked to the in-patient claims (2000–2008) to identify admissions due to breast (ICD-9-CM: 174) and endometrial (ICD-9-CM: 182) cancer. The person-year approach with Poisson assumption was used to estimate the incidence density rate. The age-specific hazard ratios (HRs) of above malignancies in relation to diabetes with multivariate Cox proportional hazard regression.

**Results:**

The overall incidence density rate of breast and endometrial cancer was estimated at 1.21 and 0.21 per 10,000 patient-years, respectively, for diabetes. The corresponding figures for controls were lower at 1.00 and 0.14 per 10,000 patient-years. Compared with the controls, the covariate adjusted HR for breast and endometrial cancer was 1.42 (95% confidence interval (CI) 1.34–1.50) and 1.71 (95% CI 1.48–1.97), respectively in women with diabetes. Elderly (> = 65 years) diabetes had the highest HR (1.61) of breast cancer, while the highest HR (1.85) of endometrial cancer was observed in diabetes aged < = 50 years.

**Conclusions:**

Diabetes may significantly increase the risks of breast and endometrial cancer in all age stratifications. Health education for strict adherence of cancer screening program in women with diabetes is essential.

## Introduction

Apart from various microvascular and macrovascular complications, accompanying insulin resistance and hyperinsulinemia of diabetes have been hypothesized to be associated with many kinds of tumor, but links with breast and endometrial cancer remain controversial. A number of studies found that women with diabetes were at increased risk of breast [1]–[8] and endometrial [1], [2], [7], [9]–[14] cancer, but not all studies revealed similar association [15]–[18]. Although recent literature review tends to support a link between diabetes and increased risk of breast [19] and endometrial [14] cancer in women, several questions remain open for investigations. First, diabetes and breast cancer association was found to be more apparent among postmenopausal women [1], [3], [6], [8], [20], but a lack of statistically significant association between diabetes and premenopausal breast cancer could be due to limited number of breast cancer patients of younger ages. Additionally, little is known about whether such menopausal difference was also observed in diabetes patients with endometrial cancer [9]. Second, a recent meta-analysis that included 16 studies published between 2000 and 2010 reported that the correlation between diabetes and breast cancer was the most obvious in Europe, followed by America. In Asia, the result was not significant [19]. Because only 3 studies were conducted in Asian populations, whether geographical distribution can affect the relationship between diabetes and breast cancer remains speculative and unproven.

In addition to the aforementioned puzzles, accompanying illness like hypertension [21], endometriosis [22], and abortion [23] that may be risk factors for breast cancer was rarely adjusted in previous studies. Moreover, none of the previous studies included Charlson’s comorbidity score [22]–[25] in their analysis to assess the severity of underlying illnesses suffered by each patient.

In Taiwan, breast and endometrial cancers are the first and seventh commonest forms of cancers in women respectively, and their incidences are increasing in recent years [26]. However, little is known about age-specific risks of breast and endometrial cancer among pre- and postmenopausal patients with diabetes. The aim of this study was to estimate the incidence and relative risks of malignant cancerof breast and endometrium among female diabetes population with various age stratifications after adjustment of Charlson’s comorbidity score and other potential clinical risk factors. Our nationally representative diabetes cohort was selected from National Health Insurance (NHI) claims in Taiwan.

## Methods

### Source of Data

Data analyzed in this study were retrospectively retrieved from the claims of the National Health Insurance Research Database (NHIRD) provided by the Bureau of National Health Insurance (BNHI). The NHIRD provides all inpatient and ambulatory medical claims for around 99% of Taiwanese people [27]. To ensure the accuracy of claim files, the BNHI performs quarterly expert reviews on a random sample for every 50 to 100 ambulatory and inpatient claims [28]. Therefore, information obtained from the NHIRD is considered to be complete and accurate [29], [30].We used several NHIRD datasets in this study, including ambulatory care visit claims (ACVC), inpatient claims (IC), and Registry for Beneficiaries (RB). Access to research data has been reviewed and approved by the Review Committee of the National Health Research Institutes.

### Study Design and Populations

This is a population-based cohort study. Details of the NHI claim data and the methods of selection of patients with diabetes and control subjects were described in our previous report [28]. Briefly, we considered a patient to be diabetes if she or he had diagnosed as having diabetes (ICD-9-CM: 250 or A-code: 181) in 2000, and again within the subsequent 12 months. To avoid accidental inclusion of miscoded patients, we further selected only those patients with the first and last outpatient visits at least 30 days apart. Additionally, we excluded those patients who were admitted to the hospitals for any malignant cancer (ICD-9-CM: 140–208) between 1997 and the date of initial ambulatory care visit for diabetes treatment in 2000. In Taiwan, major illness/injury certificates are issued to all patients with malignant cancer. In order to avoid incorrect exclusion of cancer patients, we excluded only those cancer patients with a major illness/injury certificates for an admission. For the specific purpose of this study, we limited the diabetes to females. Those aged 20 or less were also excluded to ensure that most of the diabetes were type 2. Thus, the final cohort consisted of 319,310 patients with diabetes. The date of the first outpatient visit in 2000 was the index date for each patient.

The control subjects were identified from the RB. We excluded people with claims for ambulatory care for diabetes or hospitalized for any type of malignancy (ICD-9-CM: 140–208) along with issued major illness/injury certificates between 1997 and 1999. Then we selected age- and sex-matched control subjects by using the frequency matching procedure. Because of missing information on the age or sex of 661 patients with diabetes, we could select only 614,871 control subjects. Again, we limited our control subjects to females (*n* = 319,308). The index date for subjects in the control group was their date of enrollment to NHI. If their date of enrollment was before January 1, 2000, the index date was set as January 1, 2000, which was the starting point for the follow-up for controls.

### End-points and Covariates

We used the unique personal identification number (PIN) of each insurer in both groups and linked them to the inpatient claims of 2000 to 2008 in order to identify the primary or secondary diagnoses of malignant cancer of breast (ICD-9-CM: 174) and endometrium (ICD-9-CM: 182), which were the end points of this study. To avoid incorrect assessment of malignant cancer, we included only those patients who possessed major illness/injury certificates for those admissions. The day of hospitalization of the patients was considered as the date on which the clinical endpoint of interest occurred. The study period was from January 1, 2000, to December 31, 2008.

The geographic location of each individual’s NHI unit, either the location of employment or residential area, was classified into North, Central, South, or East or into level of urbanization status (i.e., urban or rural), as per the National Statistics of Regional Standard Classification [31]. Information on a study subject’s underlying illnesses was retrieved from the inpatient and outpatient claims from the first day of 1997 to the index date of 2000. These illnesses included hypertension (ICD-9-CM: 401.9) [21], endometriosis (ICD-9-CM: 617.9) [22], and history of abortion (ICD-9-CM: 634–639) [23] in the analysis of breast cancer in relation to diabetes. On the other hand, only hypertension was considered in the analysis of diabetes in predicting onset of endometrial cancer. We calculated the Charlson’s score to indicated an individual’s level of co-morbidity. The Charlson comorbidity index is a weighted summary measure of clinically important concomitant diseases that has been adapted for use with ICD-9-CM coded administrative databases [24], [25]. We also adjusted for the frequency of outpatient visits for each study subject to avoid disease surveillance bias arising from the fact that patients with diabetes are more likely than their control counterparts to seek medical care, leading to an spuriously elevated risk of cancer in diabetes.

### Statistical Analysis

In the statistical analyses, the age-specific incidence density rate was first calculated with person-years as the denominator under the Poisson assumption. The incidence density rate was used when the denominator was the sum of the person-time values (person-years in the current study) of the at-risk population. To assess the independent effects of diabetes on the risks of breast and endometrial cancer, we used Cox proportional hazard regression models, adjusting for age, geographic area, urbanization status, Charlson’s score, and selected underlying illnesses. Taking into account both geographic area and urbanization status was made for adjustment of possible geographic variations in cancer incidence and mortality in Taiwan [32].

The study participants who encountered in-hospital mortality for causes other than cancer of breast and endometrium were considered censored from the survival analysis, and the date of censoring was the date of their deaths. If a study subject had no in-hospital mortality, the date of censoring was either the date of his/her withdrawal from NHI or the date of termination of the study, i.e., December 31, 2008. All the statistical analyses were performed using the Statistical Analysis Software (version 9.2; SAS Institute, Cary, NC). A *P* value of <0.05 was considered statistically significant.

## Results

The mean (±SD) age of patients with diabetes was 61.06 (±12.35), similar to that of control subjects (60.95±12.49). Patients with diabetes and control subjects were also comparable with respect to the distributions of geographic area and urbanization status. Compared to control subjects, patients with diabetes tended to have more co-morbid conditions. The mean Charlson’s score for diabetes and controls was 0.33±1.21 and 0.07±0.57, respectively. The characteristics of the study subjects are listed in Table 1. The median time of follow-up was 6.9 years for both the groups.

**Table 1:** Characteristics of the study subjects.

Variables	Control Group		Diabetes Group	
	n	(%)	n	(%)
**Characteristics**				
Age (years)				
20–49	55602	(17.41)	55601	(17.41)
50–64	128389	(40.21)	128389	(40.21)
≧65	135317	(42.38)	135320	(42.38)
Mean (±SD)	60.95	(±12.49)	61.06	(±12.35)
Geographic area				
Northern	139857	(43.31)	139262	(43.10)
Central	80864	(25.04)	77009	(23.83)
Southern	89545	(27.73)	93415	(28.91)
Eastern	8942	(2.80)	9624	(2.98)
Urbanization status				
Urban area	210551	(65.41)	211183	(65.51)
Rural area	108757	(33.79)	108127	(33.54)
**Comorbidity**				
Charlson’s score				
0	308685	(96.67)	274062	(85.83)
1	6387	(2.00)	22815	(7.15)
> = 2	4236	(1.33)	22433	(7.03)
Mean(±SD)	0.07	(±0.57)	0.33	(±1.21)
Hypertension	146306	45.82	192959	60.43
Endometriosis	1687	0.53	1613	0.51
Abortion	2430	0.76	2477	0.78
Mean number of ambulatory visit in 2000 (±SD)	20.9	(±18.2)	34.6	(±21.1)
Total	319308	(100.00)	319310	(100.00)

Over 9 years of follow-up, 2,945 patients with diabetes were admitted for breast cancer, and 2,656 individuals in the control group were admitted for the same diagnosis. The overall incidence densities of diabetes and controls were 1.21 and 1.00 per 10,000 person-years, respectively. For both groups, the highest incidence density rate was noted in patients aged 50–64 (1.37 and 1.17 per 10,000 person-years for diabetes and controls, respectively). After controlling for potential covariates, patients with diabetes were found to have a significantly increased risk of developing breast cancer, with an overall hazard ratio (HR) of 1.42 (95% CI 1.34–1.50). We observed a significant interaction between the diabetic status and age (*P*<0.0001), and hence, we further conducted an age-stratified analysis. The adjusted HR was most increased in elderly (> = 65 years) patients with diabetes (HR 1.61, 95% CI 1.45–1.78), followed by patients with diabetes aged 50–64 (HR 1.40, 95% CI 1.29–1.51). Younger patients (<50 years) had a relatively small but still significantly increased HR of breast cancer (HR 1.16, 95% CI 1.02–1.31) (Table 2).

**Table 2:** Overall and age-specific incidence densities and relative hazards of breast cancer (ICD-9∶174) in the diabetes and control groups.

	Control Group			Diabetes Group			Crude HR (95% CI) in association with diabetes group	Adjusted HR† (95% CI) in association with diabetes group
	No. of subjects	No. of events	ID* (per 10,000 patient-years) (95% CI)	No. of subjects	No. of events	ID* (per 10,000 patient-years) (95% CI)		
Age (years)								
20–49	55602	562	1.15(1.06–1.25)	55601	536	1.14(1.05–1.24)	0.99(0.88–1.12)	1.16(1.02–1.31)
50–64	128389	1311	1.17(1.11–1.24)	128389	1432	1.37(1.30–1.44)	1.17(1.09–1.26)	1.40(1.29–1.51)
≧65	135317	783	0.74(0.69–0.80)	135318	977	1.05(0.99–1.12)	1.41(1.28–1.55)	1.61(1.45–1.78)
Total	319308	2656	1.00(0.96–1.04)	319310	2945	1.21(1.16–1.25)	1.21(1.15–1.28)	1.42(1.34–1.50)

ID, incidence density; CI, confidence interval; HR, hazard ratio; AHR, adjusted hazard ratio.

* Based on Poisson assumption.

† Based on Cox proportional hazard regression with adjustment for age, geographic area, urbanization status, Charlson’s score, history of hypertension, endometriosis, and abortion, and frequency of medical visit.

Table 3 shows the incidence densities and HRs of endometrial cancer in both diabetes and control subjects. We observed that 520 and 372 patients from the diabetes and controls, respectively, were hospitalized for endometrial cancer between 2000 and 2008. The overall incidence density rate calculated for the diabetes and control subjects was 0.21 and 0.14 per 10,000 person-years, respectively, which represents a covariate adjusted HR of 1.71 (95% CI 1.48–1.97). Again, we observed that the incidence of endometrial cancer varied with age in patients of both groups. In patients with diabetes, the highest incidence density rate was observed in younger (<50 years) patients (0.28 per 10,000 person-years), and the lowest one was noted for the elderly (> = 65 years) patients (0.15 per 10,000 person-years). The highest incidence density rate for the control subjects was found for subjects aged 50–64 yeas (0.17 per 10,000 person-years). As compared to the control group, the diabetes group had a significantly increased risk of endometrial cancer (HR 1.71, 95% CI 1.48–1.97). The highest age-specific HR was noted in patients with diabetes aged <50 years (HR 1.85, 95% CI 1.36–2.50). The HR for patients with diabetes aged 50–64 years and those aged 65 and over was similar at 1.64 (95% CI 1.34–2.02) and 1.67 (95% CI 1.28–2.19). Despite that, the interaction between diabetes and age was not statistically significant (*P* = 0.2907).

**Table 3:** Overall and age-specific incidence densities and relative hazards of endometrial cancer (ICD-9∶182) in the diabetes and control groups.

	Control Group			Diabetes Group			Crude HR (95% CI) in association with diabetes group	Adjusted HR† (95% CI) in association with diabetes group
	No. of subjects	No. of events	ID* (per 10,000 patient-years) (95% CI)	No. of subjects	No. of events	ID* (per 10,000 patient-years) (95% CI)		
Age (years)								
20–49	55602	77	0.16(0.12–0.20)	55601	134	0.28(0.24–0.34)	1.81(1.37–2.41)	1.85(1.36–2.50)
50–64	128389	188	0.17(0.14–0.19)	128389	246	0.23(0.21–0.27)	1.40(1.16–1.69)	1.64(1.34–2.02)
≧65	135317	107	0.10(0.08–0.12)	135318	140	0.15(0.13–0.18)	1.47(1.15–1.90)	1.67(1.28–2.19)
Total	319308	372	0.14(0.13–0.15)	319310	520	0.21(0.19–0.23)	1.52(1.33–1.74)	1.71(1.48–1.97)

ID, incidence density; CI, confidence interval; HR, hazard ratio; AHR, adjusted hazard ratio.

* Based on Poisson assumption.

† Based on Cox proportional hazard regression with adjustment for age, geographic area, urbanization status, Charlson’s score, history of hypertension, and frequency of medical visit.

## Discussion

This population-based study found that diabetes increased the risks of both breast and endometrial cancer. The incidence density rate of breast cancer in Taiwanese patients with diabetes (1.21 per 10,000 patient years) was lower than those of Canadian (3.23 per 1,000 person years) [6], and Korean women diabetes (1.51 per 10,000 women) [4]. However, the increased risk of breast cancer associated with diabetes (i.e., 42%) noted in our study was comparable with those of previous studies [1]–[3], [8]. In addition, age was an apparent effect modifier in our study (*P*<0.001): the highest increased risk of breast cancer was observed in women diabetes aged >65 year, and the relative risk estimates attenuated with decreasing age. In patients with diabetes aged <50 years, the relative risk estimate was insignificant until it was adjusted by patients’ past medical histories like hypertension, endometriosis, and abortion. Many previous studies [1], [3], [6], [8] reported that diabetes would not increase breast cancer risk in premenopausal patients. But our study showed the opposite results, and such discrepancy could be attributable to the incomplete adjustment for medical illness suffered by those previous reports.

Regarding endometrial cancer, few studies assessed the incidence density rate of endometrial cancer in patients with diabetes. We found out that the incidence density rates of control subjects (0.14 per 10,000 patient years) and patients with diabetes (0.21 per 10,000 patient years) in our study were lower than the incidence density rate in Eastern Asia (10.3 per 100,000) [33]. The relative risks of endometrial cancer associated with diabetes noted in our study, however, was comparable with those reported in previous studies [1], [2], [7], [10], [11], [13]. Although not all studies [1], [9] revealed increased risk of endometrial cancer in young diabetes, a Swedish study [2] indicated that diabetes <40 years old still had higher standardized incidence ratio than those of ≥40 year old. This Swedish study, however, did not further assess the age-specific risks of patients with diabetes above 40 years. In our study, the relative risk decreased with increasing age, and the highest relative risk estimate of endometrial cancer was noted in diabetes <50 years. Although there was slight variation in age-specific relative risk estimates of endometrial cancer in diabetes, such variation was not statistically significant which provides no justification for further interpretation.

Type 2 diabetes constitutes 98.2% of all diabetes in Taiwan [34] so that the majority of the patients with diabetes in our study were likely to be type 2 diabetes. Hypothetically, the associated insulin resistance, compensatory hyperinsulinemia and increased insulin like growth factor in type 2 diabetes inhibit hepatic synthesis of sex hormone binding globulin (SHBG), stimulate ovarian synthesis of sex steroid, and consequently promote cellular proliferation and inhibition of apoptosis of breast and endometrial epithelium [35]. In addition to the endocrine effect of insulin, paracrine effect of insulin on secretion of adipokines has also been demonstrated to influence breast cancer risk and progression [36]. Deregulation of fatty acid synthase activity, chronic inflammation and oxidative stress [37] may also contribute possible pathogenic mechanism for increased risk of breast and endometrial cancers in type 2 diabetes. Our study demonstrated a higher increased risk of breast cancer in younger diabetes, which might suggest that the above possible mechanism with which diabetes may lead to an increased risk of breast cancer is more evident in younger diabetes.

Our study had several methodological strengths. First, both diabetes and control groups were collected from NHI database which is population-based. A nationwide insurance coverage also minimizes the potential for selection bias due to loss to follow-up of the study sample. Additionally, there is little likelihood of recall and information bias because all cancer patients were confirmed by major illness/injury certificates which required pathological confirmation of cancer. Second, insurance claim records provide access to the longitudinal records for geographically dispersed patients. Such a large number of study subjects also made it possible for us to make detailed age-stratified analyses particularly in very young patients. Third, we excluded patients with any type of malignance 3 years before the index date which enable us to evaluate accurate estimates of incidence and relative risk of malignant cancer of breast and endometrium. Fourth, adjustment of geographic area and urbanization statuses might have reduced such area related confounding factors. The potential confounding by prior illnesses and co-morbidities were also controlled in our study. Lastly, generalizibility of the effect size related to the relationship between diabetes and breast/endometrial cancer should be interpreted with caution as the findings are based on the Taiwanese cohort.

Despite the above strengths, several limitations were also noted in our study. First, exclusive reliance on the claim data might have caused potential misclassification bias. Although the accuracy of a single diabetes diagnosis in the NHI claim data in 2000 was reported to be 74.6% [38], we used at least two diabetes related diagnosis with the first and last visits >30 days apart, which might have reduced the possibility of disease misclassification. The control group might have also consisted of new onset or undiagnosed diabetes. Selection of breast and endometrial cancer in patients with major illness/injury certificates might have excluded some patients who had been waiting for pathological diagnosis. Such misclassification bias, however, was likely to be non-differential, which tends to underestimate rather overestimate the true relative risks [39]. Second, as we described previously, we were unable to differentiate between type 1 and type 2 diabetes in our study, which also limits specific interpretations of the study results. However, we limited the diabetes patients to those diagnosed after age 20 or older to provide further reassurance that most of the women diabetes is type 2. Third, due to limited information of Taiwan’s NHI claim data, we could not determine the BMI, duration and treatment regimens of diabetes, and other socioeconomic characteristics in our study population. Certain risk factors for breast cancer, including familial cancer history, number of delivery, and breast feeding, are also unavailable from the claim data. Failure to adjust for the above potential confounders might have biased the study results. Four, Screening or surveillance bias might be a concern in our study, because there are more frequent physician contacts for the diabetes patients. To address this concern, we adjusted for the number of ambulatory care visit made by each study subject in 2000 respectively).

In conclusion, over a 9-year follow-up period, women with diabetes in Taiwan were observed to experience significantly elevated risk of malignant cancer of breast and endometrium even after adjusting for potential confounding factors. Awareness of such relationship in both pre- and postmenopausal women is essential to diabetologists, and patients with diabetes should be well educated for strict adherence of current cancer screening program.
